# Three founding ancestral genomes involved in the origin of sugarcane

**DOI:** 10.1093/aob/mcab008

**Published:** 2021-02-26

**Authors:** Nicolas Pompidor, Carine Charron, Catherine Hervouet, Stéphanie Bocs, Gaëtan Droc, Ronan Rivallan, Aurore Manez, Therese Mitros, Kankshita Swaminathan, Jean-Christophe Glaszmann, Olivier Garsmeur, Angélique D’Hont

**Affiliations:** 1 CIRAD, UMR AGAP, Montpellier, France; 2 AGAP, Université de Montpellier, CIRAD, INRAE, Institut Agro, Montpellier, France; 3 Department of Molecular and Cell Biology, University of California, Berkeley, CA, USA; 4 Department of Crop Sciences, University of Illinois, Urbana, IL, USA

**Keywords:** *Saccharum*, sugarcane, polyploidy, hybridization, founding ancestral genome, diversity

## Abstract

**Background and Aims:**

Modern sugarcane cultivars (*Saccharum* spp.) are high polyploids, aneuploids (2*n* = ~12*x* = ~120) derived from interspecific hybridizations between the domesticated sweet species *Saccharum officinarum* and the wild species *S. spontaneum*.

**Methods:**

To analyse the architecture and origin of such a complex genome, we analysed the sequences of all 12 hom(oe)ologous haplotypes (BAC clones) from two distinct genomic regions of a typical modern cultivar, as well as the corresponding sequence in *Miscanthus sinense* and *Sorghum bicolor*, and monitored their distribution among representatives of the *Saccharum* genus.

**Key Results:**

The diversity observed among haplotypes suggested the existence of three founding genomes (A, B, C) in modern cultivars, which diverged between 0.8 and 1.3 Mya. Two genomes (A, B) were contributed by *S. officinarum*; these were also found in its wild presumed ancestor *S. robustum*, and one genome (C) was contributed by *S. spontaneum*. These results suggest that *S. officinarum* and *S. robustum* are derived from interspecific hybridization between two unknown ancestors (A and B genomes). The A genome contributed most haplotypes (nine or ten) while the B and C genomes contributed one or two haplotypes in the regions analysed of this typical modern cultivar. Interspecific hybridizations likely involved accessions or gametes with distinct ploidy levels and/or were followed by a series of backcrosses with the A genome. The three founding genomes were found in all *S. barberi*, *S. sinense* and modern cultivars analysed. None of the analysed accessions contained only the A genome or the B genome, suggesting that representatives of these founding genomes remain to be discovered.

**Conclusions:**

This evolutionary model, which combines interspecificity and high polyploidy, can explain the variable chromosome pairing affinity observed in *Saccharum*. It represents a major revision of the understanding of *Saccharum* diversity.

## INTRODUCTION

Interspecific hybridization, sometimes accompanied by polyploidization, is an important evolutionary process in plants and is associated with the domestication and/or diversification of some major crops [e.g. banana (Simmonds 1962; Perrier *et al.*, 2011), citrus (Wu *et al.*, 2014), date palm (Flowers *et al.*, 2019), rice (Santos *et al.*, 2019) and wheat (McFadden and Sears, 1946)].

Polyploids are generally divided into two categories: autopolyploids, which formed within a single species, and allopolyploids, which resulted from hybridization between two or more species. Autopolyploids are typically characterized by random association among homologous chromosomes during meiosis, leading to polysomic segregation, whereas allopolyploids have sets of homoeologous chromosomes that do not typically pair, leading to disomic segregation (Doyle and Egan, 2010). A continuum in the parental divergence of polyploids yields many intermediate situations (Stebbins, 1950; Barker *et al.*, 2016).

Polyploidy and recurrent interspecific hybridizations complicate the reconstruction of phylogenetic relationships between genera and species, particularly in higher-order polyploids, which may have a complex history of multiple allo- and/or autopolyploidization events (Fortune *et al.*, 2007; Tennessen *et al.*, 2014; Triplett *et al.*, 2014).

Sugarcane belongs to *Saccharum sensu stricto*, a genus composed exclusively of higher-order polyploid (>4*x*) species. Despite its huge economic importance, the origin of sugarcane and the evolutionary history and taxonomy of the genus *Saccharum* (Poaceae; Andropogoneae) and its species are largely unresolved (Hodkinson *et al.*, 2002; Welker *et al.*, 2015). Several close genera (*Erianthus* section *Ripidium*/*Tripidium*, *Miscanthus* section *Diandra*, *Narenga*, *Sclerostachya*) that can occasionally hybridize with *Saccharum* have been proposed by some authors to be involved in the origin of *Saccharum* and are referred to as the ‘*Saccharum* complex’ by breeders (reviewed by Daniels and Roach, 1987, Grivet *et al.*, 2006). However, molecular data, although limited so far, do not support an important direct contribution of these genera to *Saccharum* but suggest a monophyletic origin of this genus (Grivet *et al.*, 2004).

The subdivision of the genus *Saccharum* is a matter of debate (Irvine, 1999; Evans and Joshi, 2016), but a subdivision into six species is generally used by sugarcane technologists (Daniels and Roach, 1987; Grivet *et al.*, 2006). Among them, two species are wild (*S. robustum* and *S. spontaneum*); they are well differentiated but for both species the taxonomic limit and evolutionary history have been a matter of controversy (reviewed by Daniels and Roach, 1987). *Saccharum spontaneum* (2*n* = 40–128) is a highly polymorphic species with an extensive distribution from Africa to Southeast Asia. It generally has thin stalks with no or very low sugar content. *Saccharum robustum* (2*n* = 60, 80 and up to 200) is most likely native to Southeast Asia, southeast to Sulawesi, and has long thick stalks with little or no sugar. Four ‘species’ exist only in cultivation (*S. officinarum*, *S. barberi*, *S. sinense* and *S. edule*) and are considered by some authors to be horticultural groups. The most popular scenario for sugarcane domestication, among sugarcane specialists, was first established by Brandes (1956). In this scenario, sugarcane originated in New Guinea from wild *S. robustum* by human selection possibly as much as 8000 years ago, and resulted in a series of clones accumulating sugar in the stalks identified by botanists as *S. officinarum* (2*n* = 80). These cultivars were transported by humans to the Asian continent, where they hybridized with local forms of the wild species *S. spontaneum*, giving rise to a new series of cultivars better adapted to subtropical environments and to the emergence of sugar manufacturing (Daniels and Daniels, 1976). They are called *S. barberi* for cultivars from India (2*n* = 81–124) and *S. sinense* for cultivars from China (2*n* = 116–120). The interspecific origin of these two groups of formerly cultivated sugarcane was demonstrated by molecular cytogenetics (D’Hont *et al.*, 2002). *Saccharum edule* (2*n* = 60–122) is cultivated for its edible aborted inflorescence in subsistence gardens from New Guinea to Fiji and is believed to correspond to natural mutant clones from *S. robustum* (Grivet *et al.*, 2006).

The origin of modern cultivars is well documented. They are all derived from a few interspecific hybridization events performed a century ago by breeders between the formerly cultivated groups *S. officinarum* and *S. barberi* and the wild *S. spontaneum* followed by backcrossing with *S. officinarum* (Arceneaux, 1968; Daniels and Roach, 1987). They are all high polyploids and aneuploids, with around 120 chromosomes, and molecular cytogenetics studies have highlighted that 75–85 % of their chromosomes originated from *S. officinarum* and 15–25 % from *S. spontaneum*, including some chromosomes derived from interspecific recombinations (D’Hont *et al.*, 1996; Cuadrado *et al.*, 2004; Piperidis *et al.*, 2010; Huang *et al.*, 2020; Piperidis and D’Hont, 2020). Recently, a reference sequence assembly of one mosaic basic genome of sugarcane has been produced (Garsmeur *et al.*, 2018) as well as an assembly of a tetraploid *S. spontaneum* (Zhang *et al.*, 2018). However, because of its extreme genome complexity, an assembly of the polyploid genome of a cultivar has not been obtained yet.

The objectives of the present study were to gain insight into the origin and architecture of the complex genome of modern sugarcane cultivars. For this, we analysed the sequences of all 12 hom(oe)ologous haplotypes (12 BAC clones) from two distinct genomic regions of a typical modern sugarcane cultivar. This allowed us to differentiate three groups of haplotypes. To investigate the origin of the haplotypes, we exploited sequence data from accessions representative of the diversity of *Saccharum*. We showed that two groups of haplotypes were contributed by *S. officinarum* and one group by *S. spontaneum*. These results suggested that three founding genomes were involved in the origin of the *Saccharum* genus and modern sugarcane cultivars.

## MATERIALS AND METHODS

### BAC sequencing and annotation

Ten BAC clones from the sugarcane cultivar R570 library developed by Tomkins *et al.* (1999) and identified by Jannoo *et al.* (2007) as corresponding to hom(oe)ologous chromosome segments bearing the *Adh1* gene were sequenced. Mate-pair libraries of ten BAC clones were produced and sequenced using the 454 method (FLX Titanium, Roche) and assembled with Newbler (Roche). Sequences were submitted to the EMBL database under the following accession numbers (BAC clone names in parentheses; Sh, *Saccharum* hybrid): HG531786 (Sh102M23), HG531788 (Sh111P05), HG531792 (Sh172H13), HG531793 (Sh182G15), HG531794 (Sh186P07), HG531797 (Sh192N12), HG531798 (Sh206M17), HG531799 (Sh209M19), HG531802 (Sh242M02) and HG531804 (Sh245F09). Two additional hom(oe)ologous BAC clones, Sh051L01 and Sh265O22 (accession numbers AM403006 and AM403007), were previously sequenced using the Sanger method (Jannoo *et al.*, 2007).

Twelve BACs corresponding to hom(oe)ologous chromosome segments bearing the *Rpa1* gene were identified and sequenced by de Setta *et al.* (2014). BAC sequences are available from GenBank under accession numbers KF184657 to KF184973.

For all BAC clone sequences, the structure (exon–intron) and putative function of genes were automatically predicted using the GNPAnnot Community Annotation System (Guignon *et al.*, 2012) available on the SouthGreen bioinformatics platform (https://www.southgreen.fr/). Gene predictions were manually curated using Artemis software as described in Garsmeur *et al.* (2011). Genes were numbered according to Jannoo *et al.* (2007) and de Setta *et al.* (2014) for the *Adh1* and *Rpa1* regions, respectively. Large transposable elements (TEs) were annotated in the *Adh1* region as described in Garsmeur *et al.* (2011) and for the *Rpa1* region the annotation from de Setta *et al.* (2014) was updated.

### Identification of *Miscanthus* and *Sorghum* orthologous regions

*Miscanthus sinensis* and *Sorghum bicolor* orthologous regions were identified through BLASTN alignments of CDS sequences of all genes identified in the sugarcane BAC clones (16 and 6 genes for *Adh1* and *Rpa1* regions, respectively) onto scaffolds of a preliminary genome assembly of *Miscanthus sinensis* and *Sorghum bicolor* genomes (assembly v3.0.1, available at https://phytozome-next.jgi.doe.gov/). BLASTN hits were filtered with an e-value threshold of 1e−10, and for each region one *Sorghum* chromosome segment and two *Miscanthus* paralogous regions were identified. Genes were annotated as described above for sugarcane. The available *Sorghum* annotation (https://phytozome-next.jgi.doe.gov/) was compared with the *de novo* annotations to help improvement of manual curation with Artemis software.

### Global sequence comparisons

Sugarcane hom(oe)ologous BAC sequences and the *Miscanthus* and *Sorghum* orthologous regions were aligned using BLASTN. All alignments were inspected with Artemis Comparison Tools (Carver *et al.*, 2005).

### Phylogenetic analyses

Hom(eo)ologous gene sequences, including exons and introns, were aligned with MAFFT (Katoh *et al.*, 2009). Maximum-likelihood phylogenetic trees for genes shared by most haplotypes were constructed using PhyML with the GTR evolution model and the SH-like aLRT branch support with 1000 bootstrap replicates (Guindon *et al.*, 2010).

Additional phylogenetic trees were constructed using the genomic segments shared between all hom(oe)ologous BAC sequences (from gene 6 to gene 7.5 for the *Adh1* region and from gene 1 to gene 5 for the *Rpa1* region) with an alignment-free method based on *k*-mer analysis using the AAF software (Fan *et al.*, 2015): hom(eo)ologous genomic segments were split into *k*-mers of 30 bp and homopolymeric *k*-mers were discarded. A pairwise distance matrix representing the number of *k*-mers that differed between hom(oe)ologous segments was used to reconstruct phylogenetic relationships.

Trees were visualized with Seaview (Gouy *et al.*, 2010) or FigTree v1.4.4 (http://tree.bio.ed.ac.uk/software/figtree/).

### Divergence times

The number of substitutions per synonymous site (*K*_s_) was calculated between all hom(eo)ologous gene pairs belonging to three groups of haplotypes (A, B and C). Protein sequences were aligned with Clustal W (Larkin *et al.*, 2007) and PAL2NAL (Suyama *et al.*, 2006) was used to reconstruct the multiple codon alignment based on the corresponding aligned protein sequences. The *K*_s_ values were calculated with the Nei–Gojobori method implemented in PAML (Yang, 2007). This process was performed using a script available at http://github.com/tanghaibao/bio-pipeline/tree/master/synonymous_calculation/. Divergence times were estimated using the formula *T* = average *K*_s_/(2 × 6.5 × 10^–9^) (Gaut *et al.*, 1996).

### Sequence data from accessions representative of *Saccharum* species and relatives

Two types of sequence data were used: whole-genome sequence (WGS) and targeted sequence capture data (Supplementary Data Table S1). Illumina paired-ends WGS data were available for two modern cultivars (including R570), 65 *Saccharum spontaneum* accessions and one *S. officinarum* accession (Garsmeur *et al.*, 2018; Zhang *et al.*, 2018). They represent on average a coverage of 7*x* of the total genome. In addition, pre-publication access to paired-end WGS data, obtained with the Illumina NovaSeq S4 platform, for 16 *Saccharum* accessions (one modern cultivar, three *S. barberi*, two *S. officinarum*, one *S. robustum* and nine *S. spontaneum*) was provided by the Joint Genome Institute (JGI). They represent on average a coverage of 13*x* of the total genome.

Targeted sequence data were available for 304 *Saccharum* accessions and relatives (Yang *et al.*, 2019). This included sequences that mapped to 5914 sites of the *Adh1* region but none that mapped to the *Rpa1* region. We produced a second set of targeted capture sequences for 36 accessions (6 modern cultivars, 10 *S. officinarum*, 15 *S. spontaneum*, 2 *S. barberi*, 1 *S. edule* and 2 *Miscanthus* accessions). Sequencing libraries were built with 1.5 µg of DNA by accession using a protocol adapted from Kircher *et al.* (2012) and Meyer and Kircher (2010). DNAs were sheared to obtain an average of 300 bp on a Bioruptor^®^ Standard (Catalogue No. UCD-200, Diagenode, Woburn, MA). Equal amounts of 16 genomic libraries were pooled to obtain at least 500 ng of DNA. Sequence capture by hybridization was performed on each library pool according to the manufacturer’s protocol for the myBaits^®^ target capture kits (v3.02) with the custom oligonucleotide library designed by Arbor Biosciences. The regions targeted corresponded to 40 000 exons from sugarcane gene models annotated on the R570 reference sequence (Garsmeur *et al.*, 2018), including 17 476 sites from the two regions analysed in this study (*Adh1* and *Rpa1*).

### Single-nucleotide polymorphism identification

Two subsets of read data were extracted from the WGS Illumina reads. The first one corresponded to reads that have a common *k*-mer (of size 20) with the BUSCO gene sets (Seppey *et al.*, 2019) present in the monoploid sugarcane reference sequence of Garsmeur *et al.* (2018). The second subset corresponded to reads that have a common *k*-mer (of size 20) with one of the gene exons of the *Adh1* or *Rpa1* region. *In silico* Illumina reads were generated from the R570 BAC sequences with the tool art_illumina (Huang *et al.*, 2012). For the targeted capture sequences, all the reads were used. All sequences were mapped on the monoploid sugarcane reference sequence of Garsmeur *et al.* (2018).

Single-nucleotide polymorphisms (SNPs) were identified as described by Garsmeur *et al.* (2018) (https://github.com/SouthGreenPlatform/VcfHunter/). This pipeline includes the mapping of sequence data onto the reference genome and identification and quality filtration of SNPs. For each accession and at each of the sites analysed, genotypes were determined if sequencing depth was at least 30. We coded genotypes as heterozygous if the variant occurred at least twice and at ≥4 % frequency. We coded genotypes as homozygous if no polymorphism was observed or if the variant occurred a single time and to a frequency of <1 % (which we considered to be a potential sequencing error). Ambiguous cases that did not meet these criteria were coded as missing data. Note that a few SNPs detected in the R570 BACs were not detected in the R570 WGS data. They may represent sequencing errors, technical artefacts or small variations between the sequenced accessions that represent material that has been vegetatively propagated for many years.

### Multivariate analysis

Factor analyses of distances table (AFTDs) were performed for the three distinct sets of data. For accessions for which WGS data were available, the SNPs detected in the BUSCO gene set were used. For the other accessions, SNPs detected in the targeted sequence data sets were used. AFTDs were performed with the DARwin program (Perrier and Jacquemoud-Collet, 2006), using dissimilarity matrices calculated on SNPs with an in-house program, vcf2dis.1.0.py. (https://github.com/SouthGreenPlatform/VcfHunter/blob/master/README.md)

### SNP origin analyses

The origin, *S. officinarum*/*S. robustum* versus *S. spontaneum*, of the three groups of haplotypes/BACs (A, B and C) was determined using two complementary analyses. In the first analysis, SNPs specific to each of the three haplotype groups (A, B and C) were identified and their origin was inferred based on their presence/absence in the two germplasm pools (*S. officinarum*/*S. robustum* versus *S. spontaneum)*. In the second analysis, SNPs specific to the two germplasm pools were identified and their distribution in the three haplotype groups (A, B and C) was analysed. These analyses were performed with two in-house python scripts (vcf2origin_AFB.py and vcf2origin_BFA.py respectively; https://github.com/SouthGreenPlatform/sugarcane-origins/blob/master/vcf2origin_AFB.py).

An SNP was considered specific to one group of haplotypes/BACs (A, B or C) if (1) data were available for at least one haplotype for each group of haplotypes , and (2) the SNP was only found in one haplotype group. An SNP was used for origin analysis if (1) it was present in at least two accessions representative of one of the two germplasm pools and absent in all accessions of the other pool, and (2) if sequence data were available at its position for at least five *S. officinarum*/*S. robustum* accessions and five *S. spontaneum* accessions, among the accessions selected as representative of these germplasm pools. For the *Rpa1* region, since data were available for a lower number of accessions, only three *S. officinarum*/*S. robustum* were required. The same criteria were used when considering SNPs from the two germplasm pools (*S. officinarum*/*S. robustum* versus *S. spontaneum*) and examining their presence among the haplotypes/BACs. For the *Adh1* region, 45 *S. officinarum*, 8 *S. robustum* and 175 *S. spontaneum* accessions were used as representative of these species. For the *Rpa1* region, 13 *S. officinarum*, 1 *S. robustum* and 84 *S. spontaneum* were used as representative of these species (Supplementary Data Table S1). The position on the monoploid sugarcane reference sequence (Garsmeur *et al.*, 2018) of a set of 31 832 SNPs identified as specific to *S. officinarum* versus *S. spontaneum* is shown in Supplementary Data Table S2.

## RESULTS

### Comparison of two sets of 12 hom(oe)ologous haplotypes from modern cultivar R570

**Fig. 1.**
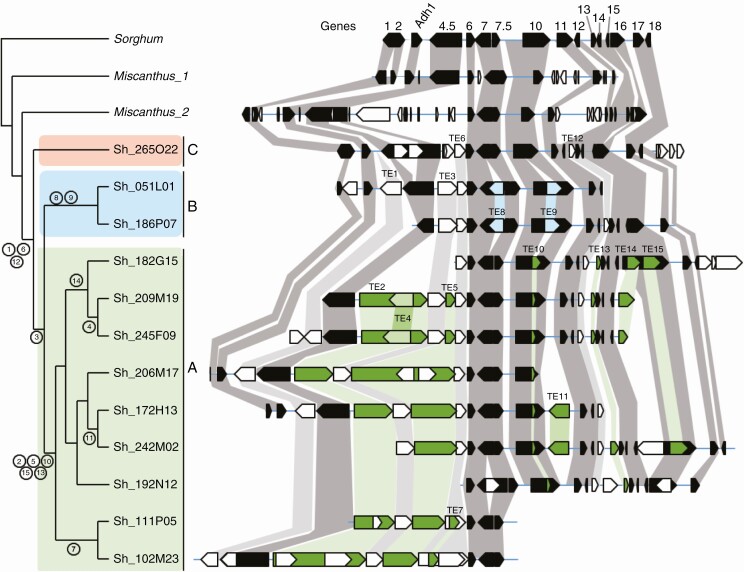
Comparison of the 12 sugarcane hom(oe)ologous haplotypes (BACs) of the *Adh1* region together and with *Sorghum* and *Miscanthus* orthologues. Genes are represented by black boxes and collinear genes are connected in dark grey. TEs are represented by white boxes; collinear TEs are connected in light grey or, when conserved within haplotype group A or B, in green or blue, respectively. Phylogenetic relationships among haplotypes are represented on the left with haplotypes from groups A, B and C highlighted in green, blue and red, respectively, and with circles positioning TE insertion times.

Two hom(oe)ologous sets of haplotypes from cultivar R570 were analysed. The first set corresponded to a region bearing the *Adh1* gene located on sugarcane chromosome 1 (Garsmeur *et al.*, 2018) and syntenic to *Sorghum* chromosome 1. This set contained 12 haplotypes, represented by 12 BAC clones. Sixteen genes with their corresponding allelic versions were annotated (Fig. 1, Supplementary Data Tables S3 and S4). The sequence that overlapped between pairs of hom(oe)ologous haplotypes varied from 35 to 113 kb, representing between 3 and 13 genes. One region of ~25 kb was shared by all hom(oe)ologous haplotypes and included three genes (genes 6–7.5).

**Fig. 2.**
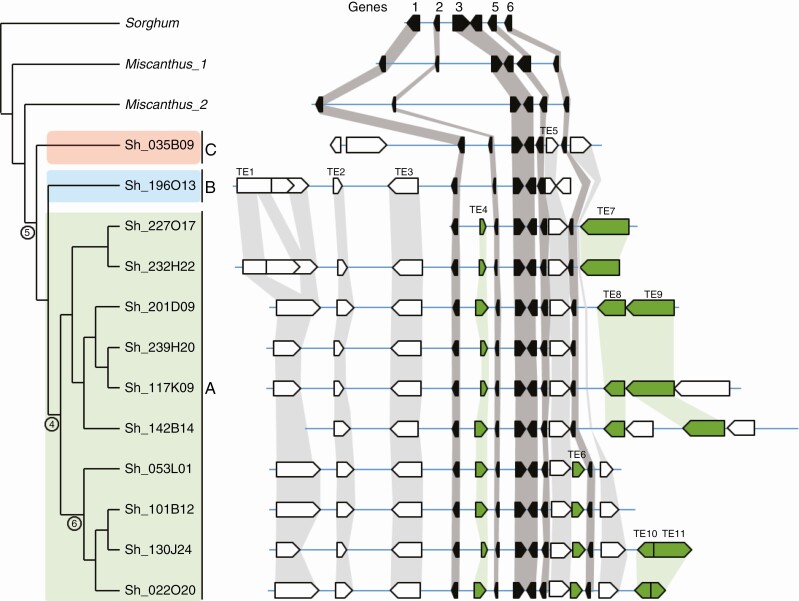
Comparison of the 12 sugarcane hom(oe)ologous haplotypes (BACs) of the *Rpa1* region together and with *Sorghum* and *Miscanthus* orthologues. Genes are represented by black boxes and collinear genes are connected in dark grey. TEs are represented by white boxes; collinear TEs are connected in light grey or, when conserved within haplotype group A, in green. Phylogenetic relationships among haplotypes are represented on the left with haplotypes from groups A, B and C highlighted in green, blue and red, respectively and with circles positioning TE insertion times.

The second set corresponded to a region bearing the *Rpa1* gene located on sugarcane chromosome 3 (Garsmeur *et al.*, 2018) and syntenic to *Sorghum* chromosome 4. This set contained 12 haplotypes, represented by 12 BAC clones. Six genes with their corresponding allelic versions were annotated (Fig. 2, Supplementary Data Tables S3 and S4). The region shared by the 12 hom(oe)ologous haplotypes included five genes and represented around 35 kb (genes 1–5).

For both regions, the gene content and relative order were strictly conserved among all hom(oe)ologous sugarcane haplotypes. The percentage of nucleotide sequence identity was very high between all pairs of sugarcane hom(oe)ologous genes, with an average of 99.2 % for exons (ranging from 97.3 to 100 %) and 95.6 % for introns (ranging from 80.3 to 100 %).

The two regions were compared with their *Sorghum* and *Miscanthus* orthologous sequences. The gene content and order were also strictly conserved between sugarcane and the *Sorghum* orthologous sequences and the two ortho-paralogous *M. sinensis* sequences (Figs 1 and 2). Both sugarcane regions displayed a high level of nucleotide sequence conservation with *Miscanthus* and *Sorghum*, with an average of 96.5 and 93.4 % for exons and 89.7 and 75.8 % for introns, respectively.

Large TEs were annotated in the *Adh1* and *Rpa1* regions, representing distinct classes of TEs [long terminal repeat (LTR) retrotransposons, non-LTR retrotransposons and DNA transposons] (Figs 1 and 2, Supplementary Data Table S4). Several TE insertion sites were conserved across hom(oe)ologous haplotypes in both regions.

### Phylogenetic relationships among hom(oe)ologous haplotypes distinguished three groups of haplotypes

**Fig. 3.**
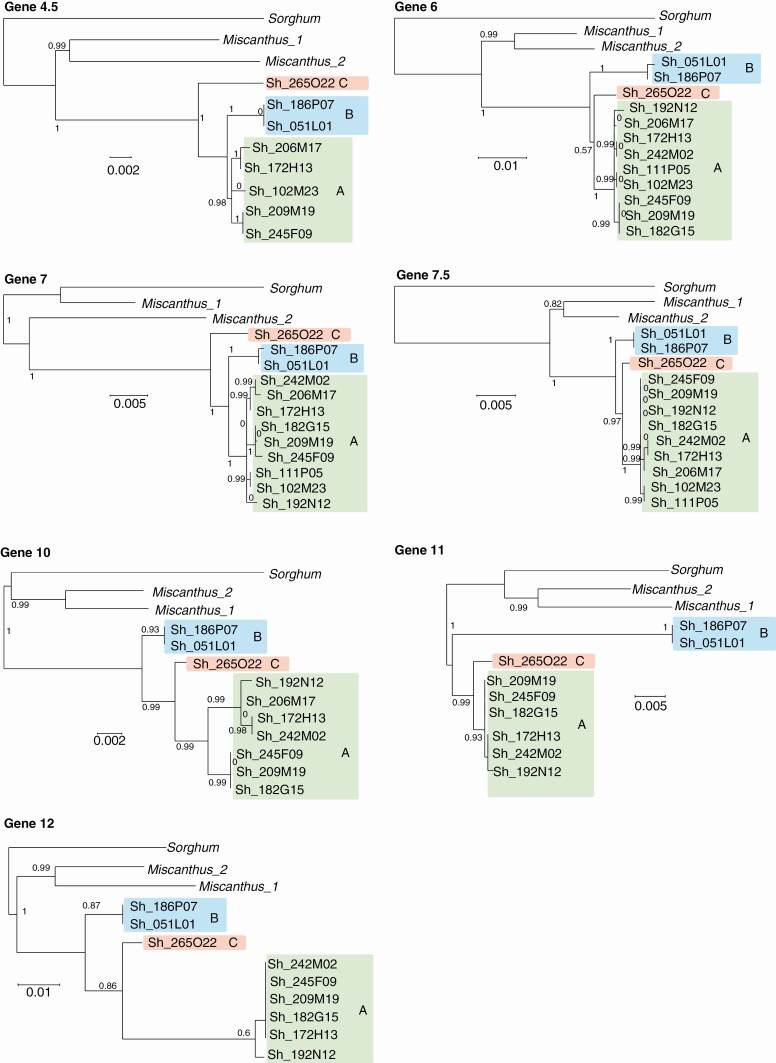
Phylogenetic relationships between hom(oe)ologous genes in the *Adh1* region. BACs belonging to haplotype groups A, B and C are highlighted in green, blue and red, respectively. Bootstrap values are indicated. Bar scales correspond to branch lengths.

**Fig. 4.**
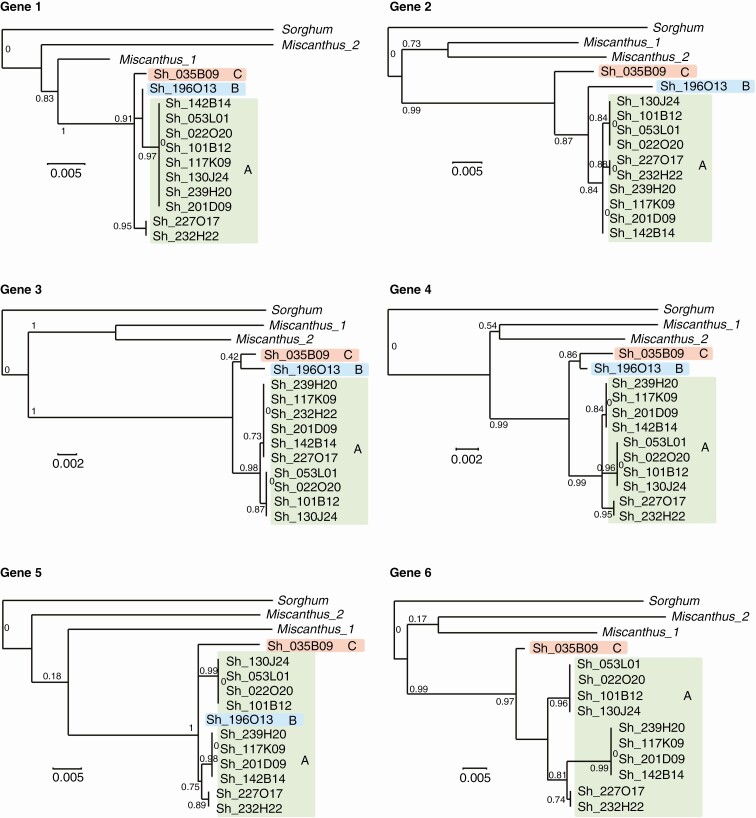
Phylogenetic relationships between hom(oe)ologous genes in the *Rpa1* region. BACs belonging to haplotype groups A, B and C are highlighted in green, blue and red, respectively. Bootstrap values are indicated. Bar scales correspond to branch lengths.

Phylogenetic relationships between hom(eo)ologous haplotypes were analysed based on (1) genomic regions shared by all haplotypes comprising genes 6–7.5 for the *Adh1* region and genes 1–5 for the *Rpa1* region (Figs 1 and 2) and (2) hom(oe)ologous copies of individual genes, including exons and introns (Figs 3 and 4).

For the *Adh1* region, both analyses revealed three groups of haplotypes, with a major group (named A) that included nine haplotypes (Sh182G15, Sh209M19, Sh245F09, Sh206M17, Sh172H13, Sh242M02, Sh192N12, Sh111P05 and Sh102M23), a second group (named B) that included two haplotypes (Sh051L01 and Sh186P07) and a third group (named C) with a unique haplotype (Sh265O22).

For the *Rpa1* region, both analyses also revealed three groups of haplotypes, in accordance with the result of de Setta *et al.* (2014), with a major group (A) that included ten haplotypes (Sh227O17, Sh232H22, Sh201D09, Sh239H20, Sh117K09, Sh142B14, Sh053L01, Sh101B12, Sh130J24 and Sh022O20) and two groups (B and C) each with a single haplotype (Sh196O13 and Sh035B09, respectively). One exception was observed for gene 1 in haplotypes Sh227O17 and Sh232H22, which grouped separately from the other A haplotypes, suggesting that recombination may have occurred in these two haplotypes.

The two *M. sinensis* paralogues grouped generally together and always apart from the group of *Saccharum* hom(oe)ologous haplotypes.

### TE insertion site conservation among hom(oe)ologous haplotypes reinforced the presence of three groups of haplotypes

In the *Adh1* region, 15 TE insertion sites were conserved across two to nine hom(oe)ologous haplotypes (Fig. 1, Supplementary Data Table S4). Two TEs were shared only by the two haplotypes of group B (TE 8 and TE 9), nine TEs were conserved only across some or all available sequences for haplotypes of group A (TE 2, 4, 5, 7, 10, 11, 13, 14 and 15) and one TE (TE 3) was shared by haplotypes of groups A and B. The TE 1, TE 6 and TE 12 insertion sites were conserved across all haplotypes overlapping the corresponding regions. For TE 1, a 15-bp sequence corresponding to the short direct repeats of this TE was found in BAC Sh265O22. This suggested that TE 1 was present on the haplotype Sh265O22 but was removed by illegitimate recombination (Ma *et al.*, 2004). The structure (complete versus fragment) was conserved among all other shared TEs, with two exceptions: TE 5 was found as a solo LTR in haplotypes Sh209M19 and Sh245F09, indicating that unequal homologous recombination between the two LTRs of the complete TE 5 occurred; TE 6 was found fragmented on haplotypes Sh111P05 and Sh102M23.

In the *Rpa1* region, in the interval that overlapped for the three groups of haplotypes, TE 4 was found on all haplotypes from group A, TE 6 on several haplotypes of group A and one TE (TE 5) was conserved on all haplotypes (Fig. 2).

Globally, the pattern of TE insertions among hom(oe)ologous haplotypes reinforced the distinction between the three groups of haplotypes, especially for the *Adh1* region, and made it possible to position the TE insertion events on the phylogenetic tree (Figs 1 and 2).

### Chronology of divergence between *Saccharum* homoeologous haplotype groups and with *Miscanthus* paralogous orthologues

Divergence times were estimated from synonymous substitution rates (*K*_s_) for each gene of both the *Adh1* and the *Rpa1* R570 region. On average, divergence times within homologous haplotypes of group A and within haplotypes of group B was low (0.05–0.34 Mya). Divergence time between haplotypes from groups A and B, from groups B and C and from groups A and C were estimated to be 0.84, 1.23 and 1.29 Mya, respectively (Table 1).

**Table 1.** Divergence time between the three groups of haplotypes (A, B and C) and between *Saccharum* and *Miscanthus*

	*K*_s_			Mya
	*Adh1*	*Rpa1*	Mean	
*Saccharum* A–A	0.0043	0.0048	0.0044	0.34
*Saccharum* B–B	0.0007	–	–	0.05
*Saccharum* A–B	0.0112	0.0102	0.0109	0.84
*Saccharum* A–C	0.0168	0.0166	0.0168	1.29
*Saccharum* B–C	0.0173	0.0129	0.0160	1.23
*Saccharum–Miscanthus*	0.0646	0.0874	0.0740	5.69
*Miscanthus 1*– *Miscanthus 2*	0.0480	0.0510	0.0513	3.94

Divergence time between *Saccharum* and *Miscanthus* lineages was estimated to be 5.7 Mya and the whole-genome duplication in *Miscanthus* to be 3.9 Mya.

### Origin of the three groups of haplotypes coexisting in modern cultivars

To determine the origin of the three distinct groups of R570 haplotypes, we exploited sequence data from accessions representative of the *Saccharum* species. These data were aligned to the R570 sugarcane monoploid reference sequence and SNPs were identified.

Because hybridization can occur between the different *Saccharum* species and because mislabelling of accessions is frequent in collections, we performed multivariate analyses for each of the three sets of sequence data (the WGS data and the two sets of targeted sequence data) to analyse the structure of the diversity within the *Saccharum* accessions and to select accessions representative of *S. spontaneum*, *S. officinarum* and *S. robustum*. For the data set from Yang *et al.* (2019), a preliminary multivariate analysis was performed to exclude accessions not belonging to the *Saccharum* genus. In this analysis, the first two axes clearly differentiated a large group of accessions labelled as *Saccharum* from scattered accessions that included accessions from closely related genera (*Miscanthus*, *Erianthu*s, *Sorghum*) and several accessions probably mislabelled as *Saccharum* (Supplementary Data Fig. 1). These accessions were excluded and the remaining 272 *Saccharum* accessions were kept for further analysis.

**Fig. 5.**
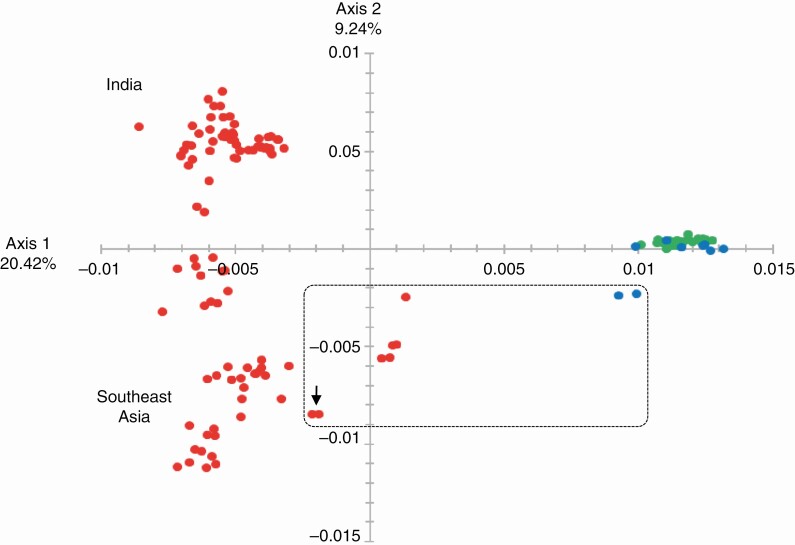
First plane of a multivariate analysis separating accessions in two germplasm pools: one pool comprises *S. officinarum* (green) and *S. robustum* (blue) accessions, and one pool comprises *S. spontaneum* (red) accessions. Analysis is based on SNPs obtained from targeted capture sequences of 142 accessions (Yang *et al.*, 2019). Accessions located between these two germplasm pools within the black rectangle may represent hybrids and were excluded for the selection of SNPs specific to these germplasm pools. Arrow points to accessions IN 84-088 and IN 84-089.

In the multivariate analyses performed for each of the three sets of sequence data only with the *Saccharum* accessions, the first axis separated *S. officinarum* and *S. robustum* accessions from *S. spontaneum* accessions. Accessions belonging to *S. barberi*, *S. sinense* and modern cultivars were in an intermediate position in accordance with their interspecific origin (Supplementary Data Figs S2, S3 and S4). Multivariate analyses were then performed without these hybrid accessions. The first axis clearly separated *S. officinarum* and *S. robustum* accessions from *S. spontaneum* accessions (Fig. 5 and Supplementary Data Figs S3b and S4). *Saccharum spontaneum* accessions formed a very large group, with the second axis largely separating accessions from India from accessions from Indonesia in the first axis of a multivariate analysis performed with targeted capture sequences of 142 accessions (Fig. 5). A few accessions labelled as *S. robustum* and *S. spontaneum* had intermediate positions. They could represent hybrids between these groups and thus were excluded from the set of accessions chosen as representatives of the three species for the selection of specific SNPs. Due to their close proximity in the multivariate analysis, *S. robustum* and *S. officinarum* accessions were further considered as one germplasm pool while *S. spontaneum* accessions were considered as a second germplasm pool for the selection of SNPs specific to each of these two germplasm pools.

Two analyses were then performed with the selected accessions to study the origin of the three groups of R570 haplotypes/BACs (groups A, B and C). In the first analysis, we identified SNPs specific to each of the three groups of haplotypes/BACs in the *Adh1* and *Rpa1* regions (i.e. SNPs found only in all or some haplotypes from a single group). A total of 188 and 60 such SNPs were identified for the *Adh1* and *Rpa1* regions, respectively. Combining the two regions, 97 SNPs were found only in haplotype group A, 65 SNPs only in haplotype group B and 86 SNPs only in haplotype group C (Table 2 and Supplementary Data Tables S5 and S6). We then identified among the accessions selected as representative of *S. robustum*/*S. officinarum* and *S. spontaneum* which one had these SNPs. SNPs specific to a haplotype group in R570 but shared by both germplasm pools were interpreted as ancestral SNPs. All SNPs specific to haplotype groups A and B and present in only one germplasm pool were found only (with one exception) in the *S. officinarum*/*S. robustum* pool, suggesting that haplotypes A and B originated from this germplasm pool. This corresponded to 57 and 25 SNPs in the *Adh1* and *Rpa1* regions, respectively (Table 2). All SNPs specific to haplotype group C and found in only one germplasm pool were found only in the *S. spontaneum* pool, suggesting that haplotype C originated from this species. This corresponded to 28 and 16 SNPs in the *Adh1* and *Rpa1* regions, respectively (Table 2).

**Table 2.** Distribution of SNPs specific to each of the three groups of haplotypes in representatives of the *Saccharum* species in regions *Adh1* and *Rpa1*

	*S. officinarum/S. robustum*	*S. spontaneum*	All three species	Total
*Adh1*				
Group				
A	29	0	44	73
B	28	1	19	48
C	0	27	40	67
Total	57	28	103	188
*Rpa1*				
Group				
A	15	0	9	24
B	10	0	7	17
C	0	16	3	19
Total	25	16	19	60

In the second analysis, from the sequences of the *Saccharum* representatives that mapped to the *Adh1* and *Rpa1* regions, we identified SNPs that were specific to each germplasm pool (i.e. SNPs found only in some or all *S. officinarum*/*S. robustum* accessions versus SNPs found only in some *S. spontaneum* accessions). A total of 96 and 44 such SNPs were identified for the *Adh1* and *Rpa1* regions, respectively (Table 3 and Supplementary Data Tables S7 and S8). We then identified which of these SNPs were present in haplotype groups A, B and C. The SNPs found only in the *S. officinarum*/*S. robustum* accessions were exclusively (with one exception) found in R570 haplotype groups A and B while the SNPs found only in *S. spontaneum* accessions were exclusively found in R570 haplotype group C.

**Table 3.** Distribution of SNPs specific to *S. officinarum*/*S. robustum* versus *S. spontaneum* in the three groups of haplotypes in regions *Adh1* and *Rpa1*.

	Group A	Group B	Group A and B	Group C	Total
*Adh1*					
* S. officinarum*/*S. robustum*	29	28	4	0	61
* S. Spontaneum*	0	1	0	27	28
* *Total	29	29	4	27	89
*Rpa1*					
* S. officinarum*/*S. robustum*	15	10	2	0	25
* S. spontaneum*	0	0	0	16	16
* *Total	15	10	2	16	43

Both analyses clearly suggested that haplotype groups A and B originated from *S. officinarum* and *S. robustum*, while haplotype group C originated from *S. spontaneum*.

### Distribution of SNPs specific to the three groups of haplotypes in *Saccharum*

The distribution of the 82 R570 SNPs specific to haplotype groups A and B that were found only in *S. officinarum*/*S. robustum* and of the 43 SNPs specific to group C found only in *S. spontaneum* was then analysed in the whole set of *Saccharum* accessions (Supplementary Data Table S1). We found SNPs specific to each of the three groups of haplotypes (A, B, C) in representatives of *S. barberi*, *S. sinense* and modern cultivars. These results are expected since they are hybrids between *S. officinarum* and *S. spontaneum* clones. SNPs specific to each of the three groups of haplotypes (A, B, C) were also found in most of the accessions from Yang *et al.* (2019) that these authors re-classified as hybrids. Many of these accessions probably corresponded to mislabelled accessions, as already suggested by Yang *et al.* (2019).

The two *S. robustum* (IN 84-076 and IS 76-184) that we excluded as representative of *S. robustum* based on the multivariate analysis displayed one SNP specific to group C in addition to SNPs specific to groups A and B, which may indicate a hybrid status. The few *S. spontaneum* clones that we excluded as representative of *S. spontaneum* displayed SNPs specific to groups A and B in addition to SNPs specific to group C, suggesting a hybrid status. Two of them (IN 84-088 and IN 84-089 in Fig. 5) positioned very close in the multivariate analysis to the other *S. spontaneum* accessions from Southeast Asia (Indonesia). These two accessions carried only SNPs specific to groups B and C except for one SNP that was specific to group A (in region *Adh1*). However, data from one of the two BACs/haplotypes of group B was missing at this position; thus the SNP could also have been present on the missing BAC/haplotype, invalidating this position as specific for group A. In addition, this SNP was found in every *S. robustum*, *S. sinense* and *S. barberi* accession and modern cultivar and in 24 out of the 28 *S. officinarum* accessions. This could indicate that this SNP is an ancestral SNP common to haplotypes of group A and B but not C, rather than specific to group A. These results suggested that these two accessions (IN 84-089 and IN 84-088) could be hybrids between the B and C ancestral founding genomes.

## DISCUSSION

We analysed 12 hom(oe)ologous haplotypes for two genomic regions in the genome of a typical modern sugarcane cultivar (R570). These regions belong to chromosomes 1 and 3, for which 12 chromosome copies were revealed with chromosome-specific oligo probes by FISH in cultivar R570 (Piperidis and D’Hont, 2020). This number is in the range of chromosome copy numbers expected for a modern cultivar (Piperidis and D’Hont, 2020). Our results revealed for both regions the existence of three groups of haplotypes, with a major group (A) being present in nine or ten copies and two minor groups (B, C) being present in one or two copies. Two wild species are known in the *Saccharum* genus, *S. spontaneum* and *S. robustum.* The sweet domesticated canes, *S. officinarum*, are thought to have been domesticated from *S. robustum* (Brandes, 1956). SNPs specific to haplotypes A and B were found in representative accessions of *S. robustum* and *S. officinarum* but not in *S. spontaneum*. Conversely, SNPs specific for haplotype C were found in representative accessions of *S. spontaneum* but not in representatives from *S. robustum* and *S. officinarum*. The three groups of haplotypes were estimated to have diverged some 0.8–1.3 Mya. These results suggested that three founding genomes were involved in the origin of the *Saccharum* genus and modern sugarcane cultivars. The observed divergence time between the three groups of haplotypes is in the range of previous estimates of the divergence between the *S. officinarum* and *S. spontaneum* lineages (0.7–3.5 Mya) (Jannoo *et al.*, 2007; Vilela *et al.*, 2017; Yang *et al.*, 2017).

The detection of specific SNPs from two groups of haplotypes (A and B) in the genomes of both *S. officinarum* and *S. robustum* is consistent with the common view that *S. officinarum* has been domesticated from *S. robustum* (Brandes, 1956). Furthermore, our study clearly demonstrated that the foundation of *S. robustum*, and hence *S. officinarum*, is heterogeneous and that interspecific hybridization or allopolyploidization occurred in the evolutionary history of this taxon in addition to autopolyploidy. The much higher proportion of haplotype A compared with haplotype B observed in both studied regions of a modern cultivar suggests that the hybridization events involved transmission of unreduced gametes or hybridizations between autopolyploids with different ploidy levels and/or were followed by backcrosses with the A founder genome.

Recently, Zhang *et al.*, 2019 contradicted the general assumption that *S. officinarum* was domesticated from *S. robustum* based on the divergence time of 0.38 Mya they estimated between assembled haplotypes from these two species. This divergence time is close to the one we obtained between haplotypes within group A (0.34 Mya). The presence of two founder genomes in the origin of these species, revealed by our study, may have complicated the interpretation of Zhang *et al.* (2019).

Specific SNPs from group C were found only in *S. spontaneum.* This species is highly polymorphic, with a large distribution range from Africa to Southeast Asia that overlaps with *S. robustum*, from Kalimantan Island to Papua New Guinea (Grivet *et al.*, 2004). The high diversity of this species is illustrated by the results of the multivariate analysis, which showed a large group with two main subgroups, one from India and one from East Asia (Fig. 5). Specific SNPs from group C but not from groups A and B were found in clones from these two *S. spontaneum* subgroups. Several clones identified as *S. spontaneum* in collections were found in intermediate positions between these *S. spontaneum* groups and the *S. robustum/S. officinarum* pool. These clones may be natural hybrids between these species since they displayed SNPs specific to groups A, B and C. These clones were found in the multivariate analysis mainly between the *S. officinarum*/*S. robustum* pool and the *S. spontaneum* accessions from East Asia (Indonesia). This can be explained by the fact that their distribution overlaps in these regions (Grivet *et al.*, 2006). Some of these *S. spontaneum* clones, based on pairwise genetic distance with modern cultivars, were suggested by Yang *et al.* (2019) to be the ones mainly involved in the origin of modern cultivars. Our analysis suggested instead that their genetic proximity with modern cultivars is linked to their hybrid status with *S. officinarum* or *S. robustum* as they bear A and B alleles specific to these species.

We found SNPs specific to each of the three groups of haplotypes (A, B, C) in representatives of *S. barberi* and *S. sinense*, in accordance with their proposed natural interspecific hybrid origin between *S. officinarum* and *S. spontaneum* (Brandes, 1956; Daniels and Roach, 1987; D’Hont *et al.*, 2002). The three groups of SNPs were also found in all modern cultivars tested, which are all derived from a few interspecific hybridizations made by breeders a century ago between a few *S. officinarum*, *S. spontaneum* and *S. barberi* clones. These interspecific hybridizations were followed by backcrosses with *S. officinarum* to recover good agronomic performance. This process resulted in the reduction of the proportion of *S. spontaneum* chromosomes, which was estimated based on molecular cytogenetics to represent between 15 and 25 % of the chromosomes in modern cultivars (D’Hont *et al.*, 1996; Cuadrado *et al.*, 2004; Piperidis *et al.*, 2010; Huang *et al.*, 2020; Piperidis and D’Hont, 2020). More recently this proportion was shown to vary from one to four copies depending on the hom(oe)ology group in the few cultivars analysed (Piperidis and D’Hont, 2020). The proportion of haplotypes A/B (11 haplotypes in the two regions) originating from *S. officinarum* versus haplotype C from *S. spontaneum* (one haplotype) observed in the two regions studied fits in this range.

This evolutionary model, implicating autopolyploid and allopolyploid/interspecific hybridization events, contradicts the assumption that *S. officinarum* has an autopolyploid origin (Garsmeur *et al.*, 2011; Vilela *et al.*, 2017; Yang *et al.*, 2017) and could explain the variable chromosome pairing affinity observed in *Saccharum* (Jannoo *et al.*, 2007). A few genetic maps have been built and all of them are partial. However, they revealed some variation in paring affinity. Some preferential pairing has been observed in *S. robustum* and *S. officinarum* (Mudge *et al.*, 1996; Edmé *et al.*, 2006; Aitken *et al.*, 2007) but not in *S. spontaneum* (Al-Janabi *et al.*, 1993; da Silva *et al.*, 1995; Alwala, 2008). This observation could suggest that *S. spontaneum* accessions are autopolyploids (from genome founder C) with polysomic pairing. This autopolyploidy was verified recently for one tetraploid clone, AP85-441 (haploid of SES 208), from which a genome sequence was assembled (Zhang *et al.*, 2018). For *S. robustum* and *S. officinarum*, the observed pairing behaviour is compatible with a mix of allo- and autopolyploid origins with the coexistence of two groups of homologues (from genome founders A and B) resulting in preferential pairing (A versus B) but each pairing being in a polysomic manner. In a modern cultivar such as R570, based on our data and Piperidis and D’Hont (2020), it is probably not rare that haplotypes B and C are present in two copies. This could explain the strong preferential pairing that we have observed for some *S. spontaneum* and some *S. officinarum* chromosomes (Grivet *et al.*, 1996; Hoarau *et al.*, 2001; Jannoo *et al.*, 2004), while the other A haplotypes largely display polysomic inheritance. Occasional recombination between homoeologues may result in mosaic chromosome structures complicating the chromosome pairing picture observed (Jannoo *et al.*, 2004).

Perfect collinearity and a very high level of gene structural conservation among hom(oe)ologous sugarcane chromosomes were observed, with an average divergence in coding sequence of <1 % and all alleles being predicted to be functional (with one exception). The striking conservation of hom(oe)ologous genes that we observed confirmed and extended our previous results on two hom(oe)ologous BACs from the *Adh1* region and seven hom(oe)ologous haplotypes from a region carrying the brown rust resistance (*Bru1*) gene (Jannoo *et al.*, 2007; Garsmeur *et al.*, 2011). One reason for this strikingly high level of gene conservation among hom(oe)ologous sugarcane haplotypes may be the relatively young age of the polyploidization event in *Saccharum* species, which may have given little time for the paralogous chromosomes to differentiate from each other. Another reason may be the high polyploidy and mixed allo- and autopolyploidy, with autopolyploidy restraining divergence through pairing and recombination between homologues and polysomic inheritance. The maintenance of a broad set of functional hom(oe)ologues could be involved in the remarkable productivity and phenotypic plasticity of sugarcane.

The comparison of the two sugarcane regions with *Sorghum* (2*n* = 2*x* = 20) orthologous regions and with the two ortho-paralogous *Miscanthus* (2*n* = 4*x* = 38) regions showed high gene synteny conservation. For both regions, gene phylogenetic analyses did not support the assumption that the allopolyploid event that arose around 3–4 Mya in the *Miscanthus* lineage after its divergence with *Sorghum* was shared with the *Saccharum* lineage (Kim *et al.*, 2014), in agreement with Welker *et al.* (2015), Vilela *et al.* (2017) and Zhang *et al.* (2018). Our results suggested that, after its divergence from the *Miscanthus* lineage, the *Saccharum* lineage differentiated in a few sublineages (A, B, C, and possibly others) that further underwent auto- and/or allopolyploid events leading to the present day higher-order polyploids (>4*x*). No extant diploid representatives of these lineages are known, presumably having become extinct. In addition, no pure representatives of the A or B lineages were found in our sample. Further investigation should be made in particular within *S. robustum*, for which we analysed only a few accessions, but these were described to display important phenotypic variation that led some authors to separate them in several distinct taxonomic groups (Daniels and Roach, 1987).

## SUPPLEMENTARY DATA

Supplementary data are available online at https://academic.oup.com/aob and consist of the following. Figure S1: first plane of a multivariate analysis based on SNPs obtained from targeted capture sequences of 304 accessions. Figure S2: first plane of a multivariate analysis based on SNPs obtained from targeted capture sequences of 272 accessions. Figure S3: first plane of a multivariate analysis based on SNPs obtained from targeted capture sequences of 33 accessions. Figure S4: first plane of a multivariate analysis based on SNPs from WGS data from 83 accessions. Table S1: information on the sequence data used. Table S2: position on the sugarcane reference sequence of Garsmeur *et al*. (2018) of 31 832 SNPs identified as specific to *S. officinarum* or *S. spontaneum* based on targeted capture sequences from 35 *S. officinarum* and 91 *S. spontaneum* accessions from Yang *et al.* (2019) Table S3: genes annotated in the *Adh1* and *Rpa1* regions. Table S4: information on TEs conserved among hom(oe)ologous chromosome haplotypes in the *Adh1* region Table S5: distribution of the SNPs specific to each of the three groups of haplotypes/BACs in the *Adh1* region in representatives of *Saccharum* diversity. Table S6: distribution of the SNPs specific to each of the three groups of haplotypes/BACs in the *Rpa1* region and in representatives of *Saccharum* diversity. Table S7: distribution of the SNPs specific to *S. officinarum*/*S. robustum* versus *S. spontaneum* in the haplotypes/BACs of the *Adh1* region. Table S8: distribution of the SNPs specific to *S. officinarum*/*S. robustum* versus *S. spontaneum* in the haplotypes/BACs of the *Rpa1* region.

mcab008_suppl_Supplementary_Figure_S1

mcab008_suppl_Supplementary_Figure_S2

mcab008_suppl_Supplementary_Figure_S3

mcab008_suppl_Supplementary_Figure_S4

mcab008_suppl_Supplementary_Table_S1

mcab008_suppl_Supplementary_Table_S2

mcab008_suppl_Supplementary_Table_S3

mcab008_suppl_Supplementary_Table_S4

mcab008_suppl_Supplementary_Table_S5

mcab008_suppl_Supplementary_Table_S6

mcab008_suppl_Supplementary_Table_S7

mcab008_suppl_Supplementary_Table_S8
