# Supplementary material for: Three founding ancestral genomes involved in the origin of sugarcane
Source: Ann Bot. 2021 Feb 26;127(6):827–40. doi: 10.1093/aob/mcab008 (PMC8103802; doi:10.1093/aob/mcab008)
Supplement: mcab008_suppl_Supplementary_Figure_S1 [file mcab008_suppl_supplementary_figure_s1.pptx]

## Slide 1
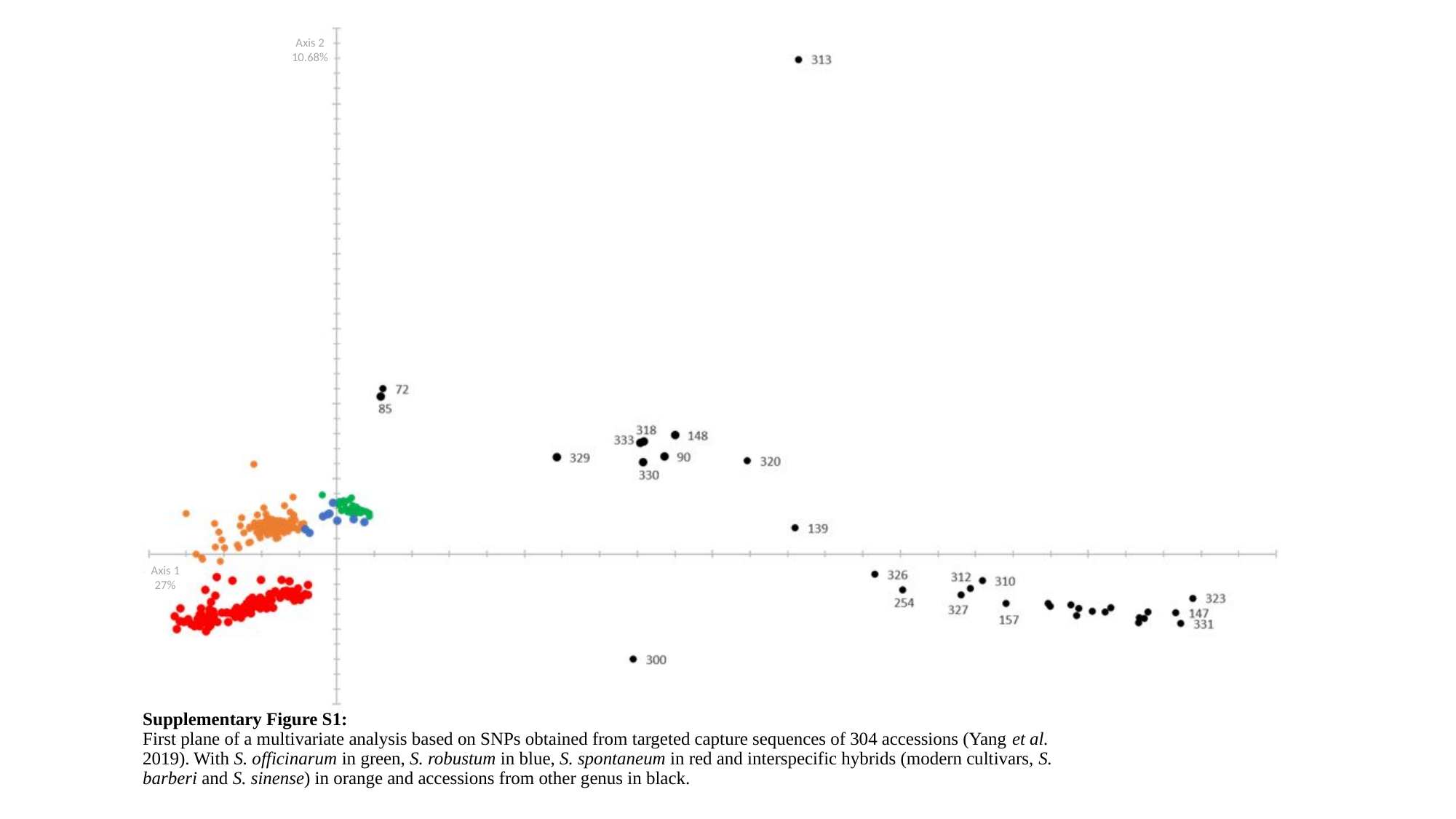

Axis 2
10.68%
Axis 1
27%
Supplementary Figure S1:
First plane of a multivariate analysis based on SNPs obtained from targeted capture sequences of 304 accessions (Yang et al. 2019). With S. officinarum in green, S. robustum in blue, S. spontaneum in red and interspecific hybrids (modern cultivars, S. barberi and S. sinense) in orange and accessions from other genus in black.
