# Supplementary material for: Three founding ancestral genomes involved in the origin of sugarcane
Source: Ann Bot. 2021 Feb 26;127(6):827–40. doi: 10.1093/aob/mcab008 (PMC8103802; doi:10.1093/aob/mcab008)
Supplement: mcab008_suppl_Supplementary_Figure_S2 [file mcab008_suppl_supplementary_figure_s2.pptx]

## Slide 1
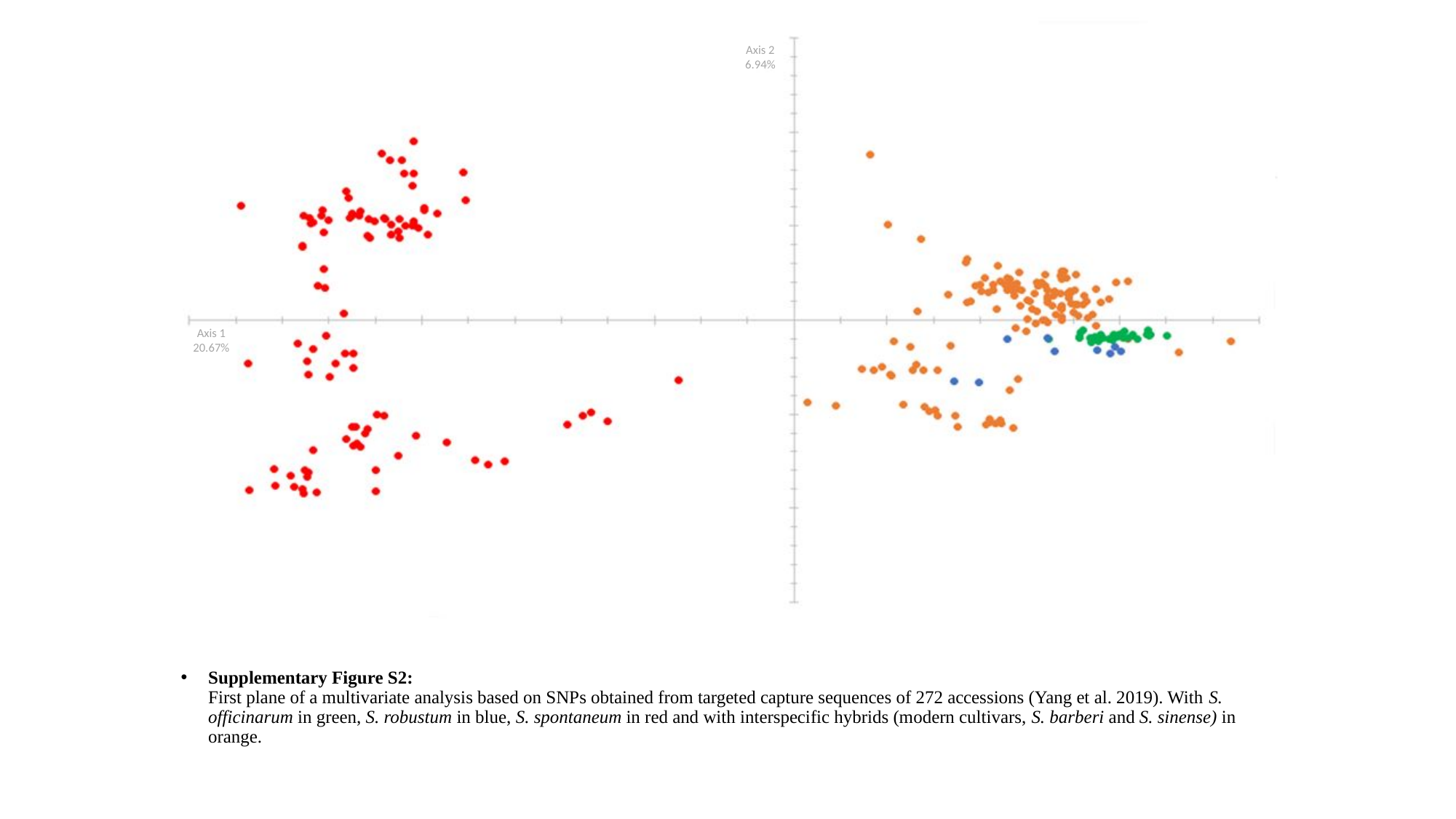

Axis 2
6.94%
Axis 1
20.67%
Supplementary Figure S2:
First plane of a multivariate analysis based on SNPs obtained from targeted capture sequences of 272 accessions (Yang et al. 2019). With S. officinarum in green, S. robustum in blue, S. spontaneum in red and with interspecific hybrids (modern cultivars, S. barberi and S. sinense) in orange.
