# Supplementary material for: Three founding ancestral genomes involved in the origin of sugarcane
Source: Ann Bot. 2021 Feb 26;127(6):827–40. doi: 10.1093/aob/mcab008 (PMC8103802; doi:10.1093/aob/mcab008)
Supplement: mcab008_suppl_Supplementary_Figure_S3 [file mcab008_suppl_supplementary_figure_s3.pptx]

## Slide 1
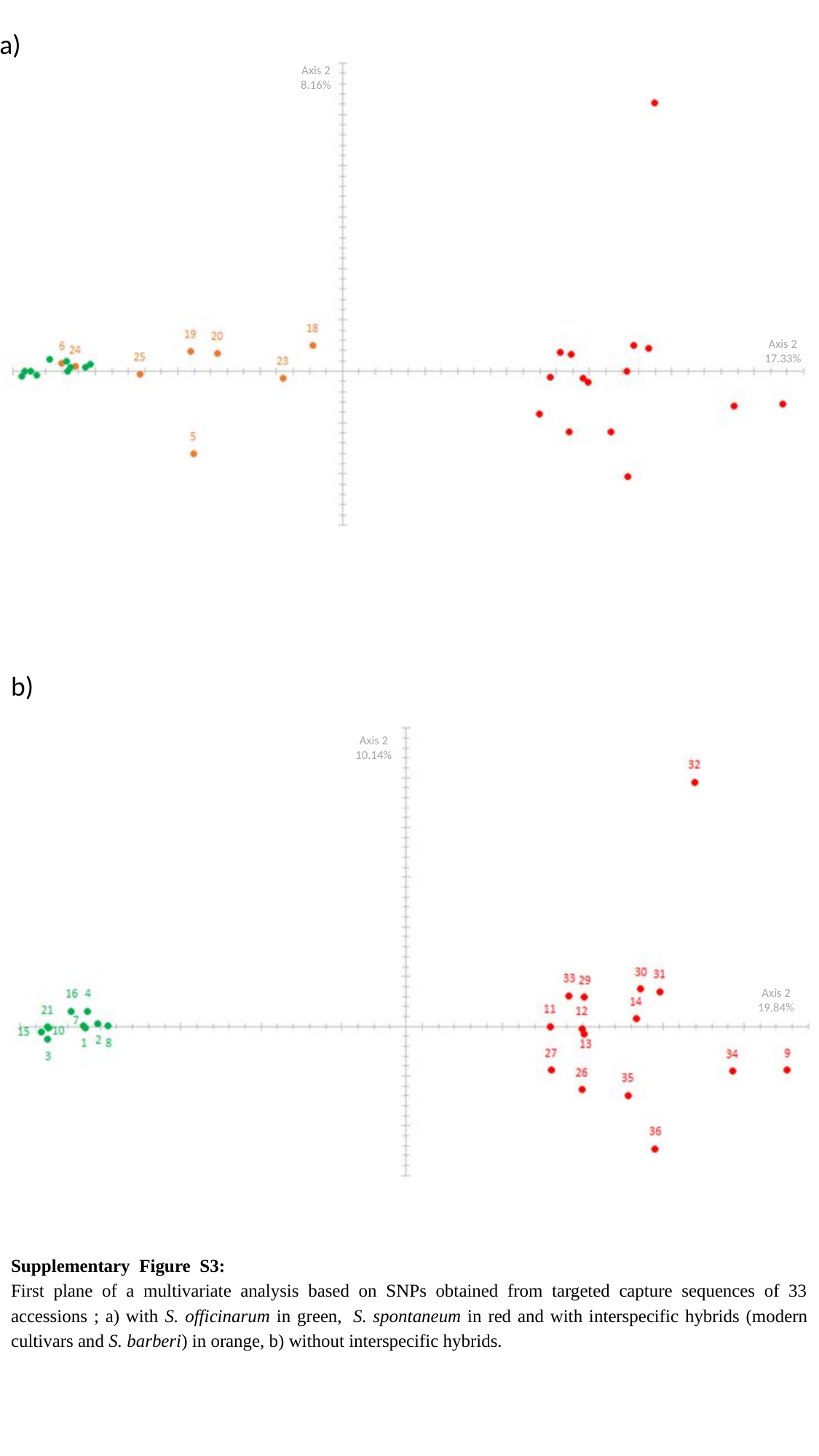

a)
Axis 2
8.16%
Axis 2
17.33%
b)
Axis 2
10.14%
Axis 2
19.84%
Supplementary Figure S3:  
First plane of a multivariate analysis based on SNPs obtained from targeted capture sequences of 33 accessions ; a) with S. officinarum in green,  S. spontaneum in red and with interspecific hybrids (modern cultivars and S. barberi) in orange, b) without interspecific hybrids.
