# Supplementary material for: Three founding ancestral genomes involved in the origin of sugarcane
Source: Ann Bot. 2021 Feb 26;127(6):827–40. doi: 10.1093/aob/mcab008 (PMC8103802; doi:10.1093/aob/mcab008)
Supplement: mcab008_suppl_Supplementary_Figure_S4 [file mcab008_suppl_supplementary_figure_s4.pptx]

## Slide 1
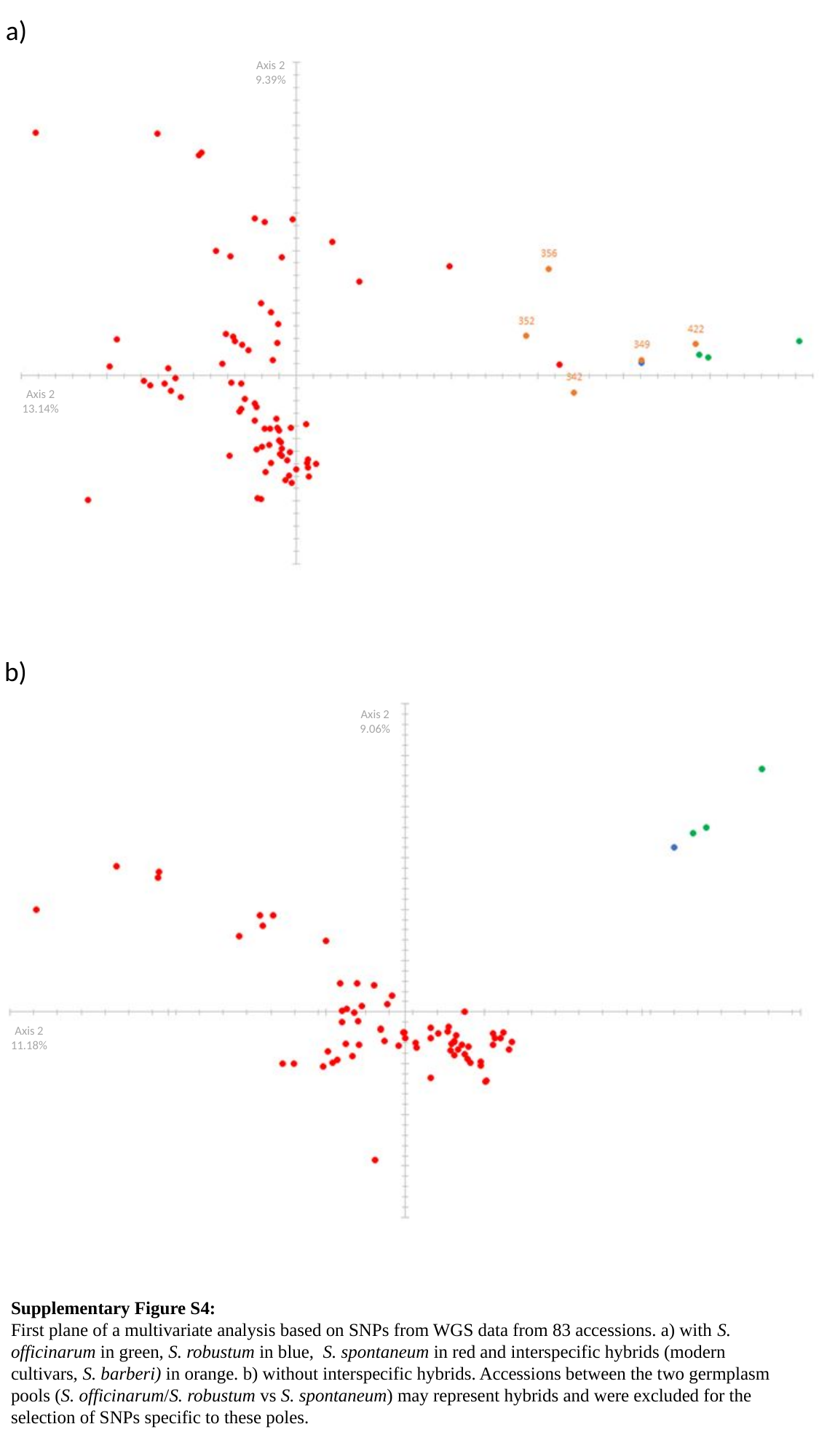

a)
Axis 2
9.39%
Axis 2
13.14%
b)
Axis 2
9.06%
Axis 2
11.18%
Supplementary Figure S4:
First plane of a multivariate analysis based on SNPs from WGS data from 83 accessions. a) with S. officinarum in green, S. robustum in blue,  S. spontaneum in red and interspecific hybrids (modern cultivars, S. barberi) in orange. b) without interspecific hybrids. Accessions between the two germplasm pools (S. officinarum/S. robustum vs S. spontaneum) may represent hybrids and were excluded for the selection of SNPs specific to these poles.
