# Supplementary material for: Three founding ancestral genomes involved in the origin of sugarcane
Source: Ann Bot. 2021 Feb 26;127(6):827–40. doi: 10.1093/aob/mcab008 (PMC8103802; doi:10.1093/aob/mcab008)
Supplement: mcab008_suppl_Supplementary_Table_S3 [file mcab008_suppl_supplementary_table_s3.doc]

**Table S3.** Genes annotated in the (a) *Adh1* and (b) *Rpa1* regions.

| **Gene*** | **Protein giving highest BLAST score (GenBank accession)** |
| --- | --- |
| 1 | Phosphatidyl-inositol 3-and 4-kinase family protein |
| 2 | Hypothetical protein |
| 3 | Alcohol dehydrogenase 1 (Adh1) |
| 4/5 | Hypothetical protein |
| 6 | Myo-inositol kinase |
| 7 | Cyclin H1 |
| 7.5 | Magnesium-dependent phosphatase 1 |
| 10 | Hypothetical protein |
| 11 | CBS domain protein |
| 12 | Hypothetical protein |
| 13 | Putative ABA-responsive protein |
| 14 | ATP synthase F1, delta subunit family protein |
| 15 | Syntaxin-related protein KNOLLE |
| 16 | Hypothetical protein |
| 17 | Endo-1,4-beta-glucanase |
| 18 | Hypothetical protein |
| 19 | Glycerol-3-phosphate acyltransferase 6-like |
| * Genes are numbered according to Jannoo et al. (2007) | |

**(a)**

**Table S3** Continued

(b)

| **Gene*** | **Protein giving highest BLAST score (GenBank accession)** |
| --- | --- |
| 1 | Nuclear transcription factor Y subunit A-8-like |
| 2 | Serine/arginine repetitive matrix protein 1-like |
| 3 | MYB-related protein |
| 4 | Replication protein A 70 kDa DNA-binding sunbunit A |
| 5 | Autophagy-related 12 isoform X1 |
| 6 | Growth-regulating factor 1-like isoform X2 |
| * Genes are numbered according to De Setta et al. (2014) | |
