# Supplementary material for: Three founding ancestral genomes involved in the origin of sugarcane
Source: Ann Bot. 2021 Feb 26;127(6):827–40. doi: 10.1093/aob/mcab008 (PMC8103802; doi:10.1093/aob/mcab008)
Supplement: mcab008_suppl_Supplementary_Table_S4 [file mcab008_suppl_supplementary_table_s4.doc]

**Table S4**. Information on TE conserved among hom(oe)ologous chromosome haplotypes in the *Adh1* region.

| **TE-insertion sites** | **Class and superfamily of TEs** | **No of sequences** | **Locus tags** |
| --- | --- | --- | --- |
| 1 | Non-LTR retrotransposon LINE | 5 | Sh051L01_te00020 |
|  |  |  | Sh102M23_te00020 |
|  |  |  | Sh172H13_te00010 |
|  |  |  | Sh206M17_te00010 |
|  |  |  | Sh245F09_te00020 |
| 2 | LTR retrotransposon Gypsy | 7 | Sh102M23_te00060 |
|  |  |  | Sh111P05_te00020 |
|  |  |  | Sh242M02_te00010 |
|  |  |  | Sh172H13_te00020 |
|  |  |  | Sh206M17_te00020 |
|  |  |  | Sh209M19_te00010 |
|  |  |  | Sh245F09_te00030 |
| 3 | LTR retrotransposon Gypsy | 7 | Sh102M23_te00080 |
|  |  |  | Sh111P05_te00040 |
|  |  |  | Sh242M02_te00020 |
|  |  |  | Sh172H13_te00030 |
|  |  |  | Sh206M17_te00030 |
|  |  |  | Sh209M19_te00025 |
|  |  |  | Sh245F09_te00045 |
| 4 | LTR retrotransposon Gypsy | 2 | Sh209M19_te00020 |
|  |  |  | Sh245F09_te00040 |
| 5 | LTR retrotransposon Gypsy | 7 | Sh102M23_te00081 |
|  |  |  | Sh111P05_te00050 |
|  |  |  | Sh242M02_te00030 |
|  |  |  | Sh172H13_te00040 |
|  |  |  | Sh206M17_te00040 |
|  |  |  | Sh209M19_te00027 |
|  |  |  | Sh245F09_te00050 |
| 6 | Non-LTR retrotransposon LINE | 8 | Sh102M23_te00085 |
|  |  |  | Sh111P05_te00055 |
|  |  |  | Sh206M17_te00065 |
|  |  |  | Sh182G15_te00010 |
|  |  |  | Sh209M19_te00030 |
|  |  |  | Sh245F09_te00060 |
|  |  |  | Sh242M02_te00032 |
|  |  |  | Sh172H13_te00042 |

**Table S4** Continued

| **TE-insertion sites** | **Class and superfamily of TEs** | **No of sequences** | **Locus tags** |
| --- | --- | --- | --- |
| 7 | LTR retrotransposon Gypsy | 2 | Sh102M23_te00090 |
|  |  |  | Sh111P05_te00060 |
| 8 | LTR retrotransposon Copia | 2 | Sh051L01_te00040 |
|  |  |  | Sh186P07_te00020 |
| 9 | LTR retrotransposon Copia | 2 | Sh051L01_te00050 |
|  |  |  | Sh186P07_te00030 |
| 10 | LTR retrotransposon Copia | 7 | Sh192N12_te00020 |
|  |  |  | Sh242M02_te00040 |
|  |  |  | Sh172H13_te00050 |
|  |  |  | Sh206M17_te00070 |
|  |  |  | Sh182G15_te00020 |
|  |  |  | Sh209M19_te00040 |
|  |  |  | Sh245F09_te00070 |
| 11 | DNA transposon | 2 | Sh242M02_te00050 |
|  |  |  | Sh172H13_te00060 |
| 12 | DNA transposon | 8 | Sh186P07_te00040 |
|  |  |  | Sh265O22_te00050 |
|  |  |  | Sh192N12_te00025 |
|  |  |  | Sh242M02_te00055 |
|  |  |  | Sh172H13_te00070 |
|  |  |  | Sh182G15_te00030 |
|  |  |  | Sh209M19_te00050 |
|  |  |  | Sh245F09_te00080 |
| 13 | DNA transposon | 5 | Sh192N12_te00035 |
|  |  |  | Sh242M02_te00056 |
|  |  |  | Sh182G15_te00040 |
|  |  |  | Sh209M19_te00060 |
|  |  |  | Sh245F09_te00090 |
| 14 | LTR retrotransposon Gypsy | 3 | Sh182G15_te00050 |
|  |  |  | Sh209M19_te00070 |
|  |  |  | Sh245F09_te00100 |
| 15 | Non-LTR retrotransposon LINE | 2 | Sh242M02_te00070 |
|  |  |  | Sh182G15_te00060 |
